# IMPROVING SUPPORT FOR CAREGIVERS OF SERIOUSLY ILL OLDER VETERANS: STEPS TOWARD LAY NAVIGATION

**DOI:** 10.1093/geroni/igz038.2508

**Published:** 2019-11-08

**Authors:** Nathan A Boucher

**Affiliations:** Durham VA Medical Center, Health Services Research & Development, Durham, North Carolina, United States

## Abstract

This session will discuss mid-stage findings from a five-year, federally-funded study to develop lay navigation supporting informal caregivers, often family/friends, of older Veterans with advanced stage illness. Caregivers of Veterans report numerous burdens in their caregiver role related to food, clothing, shelter, utilities, and transportation. Current programs focus on Veterans’ needs rather than caregivers’ needs. Few programs focus on practical needs that can be met with VA and community-based supports. Lay navigator programs may be used to support caregivers’ social/practical needs. Lay navigation is used with patient populations, but models focused on caregivers do not readily exist. Dr. Boucher will discuss establishment of and input from the study’s Stakeholder Advisory Board and data from sample of caregiver and Veteran interviews informing a lay navigation training curriculum and pilot intervention. Feedback from audience members will be encouraged in this session exploring quality improvements in caregiver support applicable to multiple health systems.

